# TLR9 regulates NLRP3 inflammasome activation via the NF-kB signaling pathway in diabetic nephropathy

**DOI:** 10.1186/s13098-021-00780-y

**Published:** 2022-02-04

**Authors:** Jinfeng Shen, Zaiyou Dai, Yunsheng Li, Huiping Zhu, Lijin Zhao

**Affiliations:** Department of Nephrology, The First People’s Hospital of Wenling, No. 333, Chuanan South Road, Wenling, 317500 Zhejiang China

**Keywords:** Diabetic nephropathy (DN), Db/db mouse, TLR9, NF-kB, NLRP3

## Abstract

**Background:**

Toll-like receptors (TLRs) are critical sensors for the conservation of bacterial molecules and play a key role in host defense against pathogens. The effect of TLRs on the maintenance of diabetic nephropathy (DN) and resistance to infection has been investigated; however, the detailed effects of TLR9 on DN development remain elusive.

**Methods:**

We performed quantitative reverse transcription-polymerase chain reaction and western blotting to detect TLR9 expression levels in the kidneys of experimental mice (db/db) and high-glucose-treated mouse mesangial cell strains (MCs).

**Results:**

TLR9 expression was found to be remarkably upregulated in the kidneys of experimental mice (db/db) and MCs cultivated under hyperglycemic conditions. Moreover, knockdown of TLR9 could restrain NF-kB viability and downregulate the NLRP3 inflammasome in high glucose-treated MCs. TLR9 inhibition also alleviated inflammation and apoptosis, which was reversed by the addition of the NF-κB activator, betulinic acid. Furthermore, depleted TLR9 levels restrained NF-κB viability and NLRP3 expression and reduced kidney inflammation, microalbuminuria discharge, blood sugar level, and glomerular damage in experimental mice (db/db) kidneys.

Conclusions

These findings offer novel insights into the regulation of TLR9 via the nuclear factor-kB/NOD-, LRR-, and pyrin domain-containing protein 3 inflammasome inflammation pathways in DN progression.

## Background

Diabetic nephropathy (DN) is an urgent public health challenge, and the most frequent pathogenesis of chronic kidney disease (CKD). It can result in premature death and end-stage renal disease [1]. Over the past few years, numerous researchers have proposed that inflammatory pathways play a central role in DN progression. Several proinflammatory molecules (e.g., interleukin-18, tumor necrosis factor-alpha, MYD88, and intercellular adhesion molecule-1) have abnormal expression levels and are strongly linked with DN progression according to clinical research and experimentation in animals [2–6].

Nuclear factor kappa B (NF-kB) plays a significant role in the DN inflammatory stage [7]. Interleukin-18, tumor necrosis factor-alpha, MYD88, and intercellular adhesion molecule-1 can be processed through transcription by NF-kB, and they each participate in the progression of diabetic glomerular damage (DGD), including changes in the extracellular matrix (ECM), glomerular sclerosis, and fibration [8]. The transcription factor NF-kB is a significant DN inflammatory-stimulating factor [8]. When nuclear factor-κB inhibitor-α (IkBα) is degraded by the immune proteasome, IkBα causes nuclear factor-kB p50/p65 heterodimer activation. The p65 subunit translocates to the cytoblast and binds to specific promoter sequences of target genes [9, 10]. Increasing number of studies are showing that NOD-, LRR-, and pyrin domain-containing protein 3 inflammasomes directly affect kidney inflammation, resulting in the development of DGD (e.g., glomerular-fibration, ECM hyperplasia, or glomerular sclerosis) [11–14]. Prior studies have also suggested that NLRP3 and NF-kB inflammasomes are involved in the inflammatory stage of numerous illnesses (e.g., DN) [15]. Consequently, the NF-kB/NLRP3 inflammasome signaling pathway may have significant effects on DN. However, the mechanisms underlying DN progression remain unclear.

Inflammation is a key pathological process in DN. Recent studies have provided insights into congenital immune system activation and induction of the proinflammatory cascade via toll-like receptor (TLR) activation. Specifically studying TLR4 and TLR2 in diabetes and its complications has led to significant interest in their key signaling mechanisms. Ligand-induced signaling of TLRs ultimately causes the activation of NF-kB, a transcription factor central to mediating inflammation pathways, thereby leading to kidney fibration and renal insufficiency. In the kidney, TLRs are differentially distributed within tubular epidermal cell strains and mesangial cell strains and can be categorized according to their subcellular localization. TLR1/2/4/5/6 are present on the cell surface, while TLR3/7/8/9 are intracellular. Thus, strategies targeting TLR-mediated inflammatory responses could potentially offer new treatment methods for DN. In this study, we aimed to provide a brief overview of TLRs in the kidney, with a particular focus on the role of TLR9 and NF-kB/NLRP3 inflammasomes in the mediation of inflammation and cellular death in DN.

## Methods

### Participants

The participants included in this investigation were admitted to the Nephrology Department of the First People's Hospital of Wenling University between July 2018 and June 2020. Patients and controls were of Han nationality and were between 36- and 56-years old. Based on the reference stage of DN diagnosis highlighted by Mogensen et al., early diagnosis of DN was performed [16]. The urinary albumin excretion rate (UARE) is one marker of the stage ((20 mg per min) ≤ UARE ≤ 200 mg per min), and two UARE tests were carried out within 6 months. Participants with the following conditions were excluded: cancer, type I diabetes mellitus (DM), complicated diabetic ketoacidosis (DKA), acute infections, cardiac-cerebral vascular illnesses, surgical operations, and CKD. Those who had taken supplementary vitamins, glucocorticoids (GC), PPARγ-activating agents, or α-lipoic acid drugs within the last 2 months were also excluded; this is because these drugs can regulate the activation of nuclear factor-kB [17]. The control group comprised healthy adults without any signs of chronic disease, DM, CKD, or liver, thyroid gland, or any other metabolic illnesses. This study was approved by the ethics committee of the First People's Hospital of Wenling and was implemented in accordance with the Declaration of Helsinki. All study participants provided written informed consent before participating in the study.

### Cell culture

Primary mouse mesangial cell strains (MCs), which were isolated and cultured as described previously [18], were placed in Dulbecco’s modified Eagle’s medium and 10% fetal bovine serum (Gibco, New York, USA) for growth. MCs were stimulated with D-glucose (D-GLU) at < 5.5 mmol per liter GLU (low-GLU or control group) or at 25 mmol per liter GLU (high-GLU or HG group). All cell strains were placed in an atmosphere containing 5% CO_2_ at 37 $^\circ$C.

### Animals

BKS.Cg-m + Leprdb/ + Leprdb/J (db/db) mice (4-week, male) and HbAHbS male mice (BKS.Cg-m + / + Leprdb/J (db/m) mice) from a single nest were provided by VitalRiver (Beijing, China). They were classified as DN mice (db/db) and control mice (db/m) following a comparison of the two groups [5]. Mice were divided into four groups: db/m (n = six), db/db-untreated (db/db, n = six), db/db-shRNA NC (n = six), and db/db-shRNA-TLR9 (n = six). The db/db-shRNA-NC and db/db-shRNA-TLR9 mice were administered lentiviral shRNA-NC (RiboBio, Guangzhou, China) and lentiviral shRNA-TLR9 (RiboBio), respectively, via tail injection once every 2 days. A dose of 10 mg/kg/day was continuously administered for 4 weeks until the urine MCA expression level in the db/db-shRNA-TLR9 group was consistently lower than that in the untreated db/db group. At 26-weeks old, all mice were euthanized via cervical dislocation under gas anesthesia (IsoFlu), and their kidneys were collected. Every procedure was approved by the Animal Care and Use Committee of Beijing VitalRiver Animal Laboratory.

### Measurement of urine albumin, blood glucose, and renal function

During the last three days of treatment, the mice were placed into separate metabolic cages. After 3 h of acclimation, urine specimens were collected for 24 h. Animals were continuously administered free H_2_O and a standard laboratory diet. The 24 h urine specimens were stored at − 80 °C until analysis. A mouse albumin enzyme-linked immunosorbent assay **(**ELISA) kit (ASABASYPRO, USA) was used to measure the UALB concentration, according to the manufacturer’s instructions.

After fasting for 12 h, Chloraldurat was administered to sedate the mice, and the blood was collected from the caudal vein. A blood sugar meter (LifeScan OneTouch Ultra, Milpitas, California, USA) was used to measure the blood sugar level of each mouse in duplicate. Serum creatinine (SCR) and blood urea nitrogen (BUN) levels were measured using an automatic analyzer (model 7170; Hitachi Co., Ltd., Japan).

### Histologic assessment

After the experimental animals were euthanized, their kidneys were quickly removed and weighed, and the renal cortex was isolated. The left cortex was deepened for western blotting (WB) and reverse transcription-polymerase chain reaction (RT-PCR) analysis, while the right cortex was retrieved for histological evaluation of kidney disease. The right cortex was fixed in 10% paraformaldehyde and embedded in paraffin to create three micron-thick slices. The slices were stained with periodic acid-Schiff (PAS). All histological assessments were performed blind. Twenty glomeruli from each mouse were assessed. The semi-quantitative scoring method was applied to evaluate the degree of injury to each glomerulus: level 0, normal glomerulus (no damage); level 1, mesentery expansion area, up to 25% (minimum damage); level 2, 26–50% swelling (moderate damage); level 3, 51–75% swelling (moderate-severe damage); level 4, 76–100% swelling (severe damage). The glomerular matrix expansion index (GMI) was calculated as described by Lu et al. [19].

### Immunohistochemistry (IHC)

IHC analysis was carried out to determine the levels of TLR9, P-NF-kB, and NLRP3 in the paraffin-embedded kidney tissue slices using several antibodies (TLR9 antibody, ab134368, Abcam; P-NF-κB antibody, ab264271, Abcam; NLRP3 antibody, ab4207, Abcam). Antihelion recovery based on pepsinum was also carried out. Based on the uniformity of the targeted-protein dyeing, computer morphometrics (Image Pro Plus 6.0, Bethesda, MD) were employed to measure protein interstitial staining. Following 400 cycles of amplification, the dyed areas of the outer medulla and cortex in 20 stochastically-selected areas were quantified as a percentage of the overall measurement area. IHC appraisal was conducted by an observer blinded to the investigation group.

### Western blotting

Cellular lysates were obtained by lysis of the cell strains in RIPA buffer. After the concentration was determined, the proteins were separated using 10% sodium dodecyl sulfate–polyacrylamide gel electrophoresis and transferred to a polyvinylidene diflouride (0.45 µm) membrane. The membrane was then blocked with BSA (5%) at 25 °C for 1 h. Using the intended antibodies, the membrane was cultured at 4 °C for approximately 16 h. Thereafter, the secondary antibodies were applied (4 °C; 1 h). The antibodies used in this experiment were: TLR9 antibody (ab134368, Abcam), P-NF-kB antibody (ab264271, Abcam), NF-kB antibody (ab16502, Abcam), NLRP3 antibody (ab4207, Abcam), Caspase-1 antibody (ab138483, Abcam), IL-1 antibody (ab9722, Abcam), PYCARD antibody (ab180799, Abcam), LaminB1 antibody (ab16048, Abcam), Collagen IV antibody (ab19808, Abcam), Fibronectin antibody (ab2413, Abcam), Bak antibody (ab104124, Abcam), Bax antibody (ab53154, Abcam), Bcl-xl antibody (ab32370, Abcam), Bcl-2 antibody (ab59348, Abcam), and Actin antibody (ab8227, Abcam). Based on the C-DiGit® Blot Scanner, a strengthened chemiluminescent reaction (SuperSignal®West Femto Maximum Sensitivity Substrate Kit, Thermo Fisher Scientific, Waltham, Massachusetts, USA) was conducted in order to visualize the blots.

### ELISA

The supernatant of cell cultures was used to detect secreted IL-6, IL-18, and TNF-α levels using ELISA kits (R&D Systems, Minneapolis, MN, USA). Absorbance was detected at 105 nm using a Bio-Rad Model 680 microplate reader (Bio-Rad, Hercules, CA, USA). The concentrations of each protein were determined from the standard curve according to the method [20].

### Real-time PCR

Trizol reagent was used to extract ribonucleic acid from cell strains. Using the Light Cycler® 480 RT-PCR system (Roche, Basel, Switzerland), the SYBR Green master mix was used to measure the mRNA levels of different genes with GAPDH used as an internal control. Quantitative RT-PCR was carried out on the Light Cycler® 480 RT-PCR system (reaction volume: 20 µL). PCR was performed under the following cycling conditions: 95 °C for 10 min, 40 cycles at 60 °C for 15 s, and 40 cycles at 72 °C for 30 s. The expression level of the target gene was confirmed using the comparative CT method (2^−ΔΔCT^) with the internal reference. The primer sequences used were as follows: IL-1β F: 5′-TGA AAT GCC ACC TTT TGA CAG-3′, IL-1β R: 5′-CCA CAG CCA CAA TGA GTG ATA C-3′; IL-6 F: 5′-TGC CTT CTT GGG ACT GAT-3′, IL-6 R: 5′-CTG GCT TTG TCT TTC TTG TT-3′; IL-8 F: 5′-GAG AGT GAT TGA GAG TG-3′, IL-18 R: 5′-CAC AAC CCT CTG CAC C-3′; and GAPDH F: 5′-GGA AAG CTG TGG CGT GAT-3′, GAPDH R: 5′-AAG GTG GAA GAA TGG GAG TT-3′.

### Cellular death analysis

The cells were assessed using an Annexin V-FITC and propidium iodide (PI) Apoptosis Detection Kit (Invitrogen, Carlsbad, CA, USA). Pulmonary microvascular endothelial cell strains were digested and rinsed with cold phosphate-buffered saline. The cell strains (1 × 10^6^ cells/mL) were resuspended in binding buffer (100 µL) containing Annexin V and PI and then cultured at room temperature for 20 min in the dark. The number of apoptotic cell strains was detected using one FC (BD Biosciences, San Jose, CA, USA).

### Statistical analysis

The results of this study are presented as means ± S.E.M. The one-way ANOVA (with Tukey’s test for post-hoc analysis) was applied to compare discrepancies between multiple groups, and the discrepancies between each two groups were determined using two-tailed t-tests. *P* < 0.05 indicates a statistically significant difference.

## Results

### TLR9 expression is upregulated in DN

**Fig. 1:**
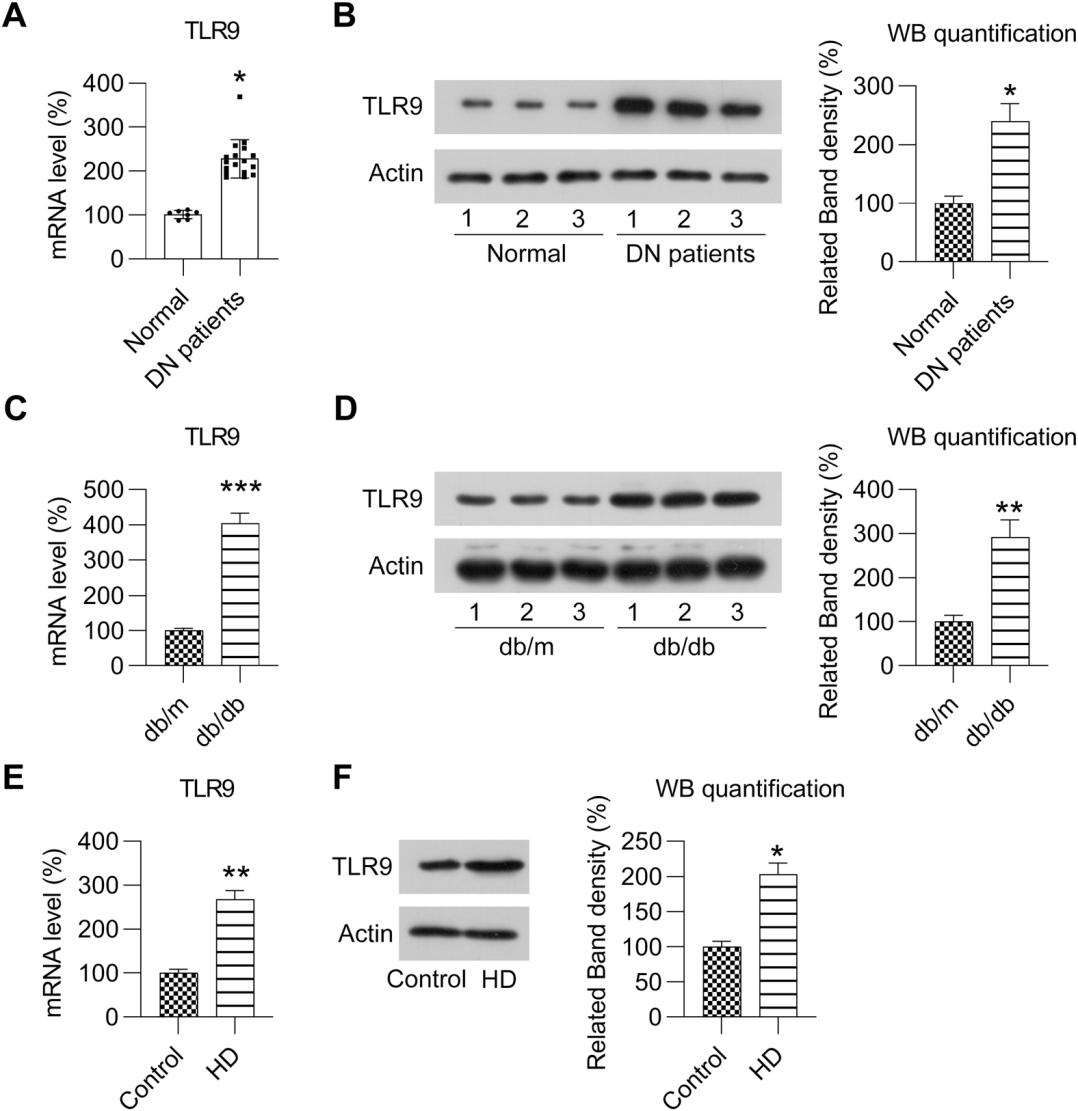
Expression of TLR9 in DN. **A** Real-time PCR revealed TLR9 expression in PBMCs from patients (healthy = 7 and DN patients = 17). **B** WB revealed protein levels of TLR9 in PBMCs from patients (healthy = 3 and DN patients = 3). **C** Real-time PCR revealed TLR9 mRNA expression in the kidneys of mice (db/db) with DN (n = 6) and mice (db/m) (n = 6) at 12 weeks. **D** WB revealed TLR9 protein levels in the kidneys of mice (db/db) with DN and control mice (db/m) at 12 weeks. **E** MCs were treated with HG for more than 12 h. RT-PCR was used to determine the level of TLR9 mRNA expression in the MCs treated with HG or normal LG. **F** WB was used to determine the protein levels of TLR9 in the MCs treated with HG or normal LG. The data are representative of the results of three independent experiments, and the data are presented as means ± S.E.M (*P < 0.05, **P < 0.01, ***P < 0.001)

To elucidate the effect of TLR9 on DN, RT-PCR and WB were conducted to determine the level of TLR9 expression in the kidney tissue of patients with DN and animal models and in cell strains. In the PBMCs of participants with DN, TLR9 expression was found to be upregulated relative to that of healthy controls at both the mRNA and protein levels (Fig. 1A, B). In the kidneys of experimental mice (db/db) with DN, the mRNA and protein expression levels of TLR9 were notably increased relative to that of control mice (Fig. 1C, D). High-blood sugar is one of the main stimulating factors for the development of kidney disease in patients with DM [21]. Accordingly, MCs were treated with high glucose to mimic a DN cell model. In this model, TLR9 expression was found to be promoted by high glucose at both the mRNA and protein levels (Fig. 1E, F). Such findings suggest that TLR9 could be induced by high sugar levels and might be related to the development of DN.

### Effects of TLR9 knockdown on blood glucose and renal function in experimental mice (db/db)

**Fig. 2:**
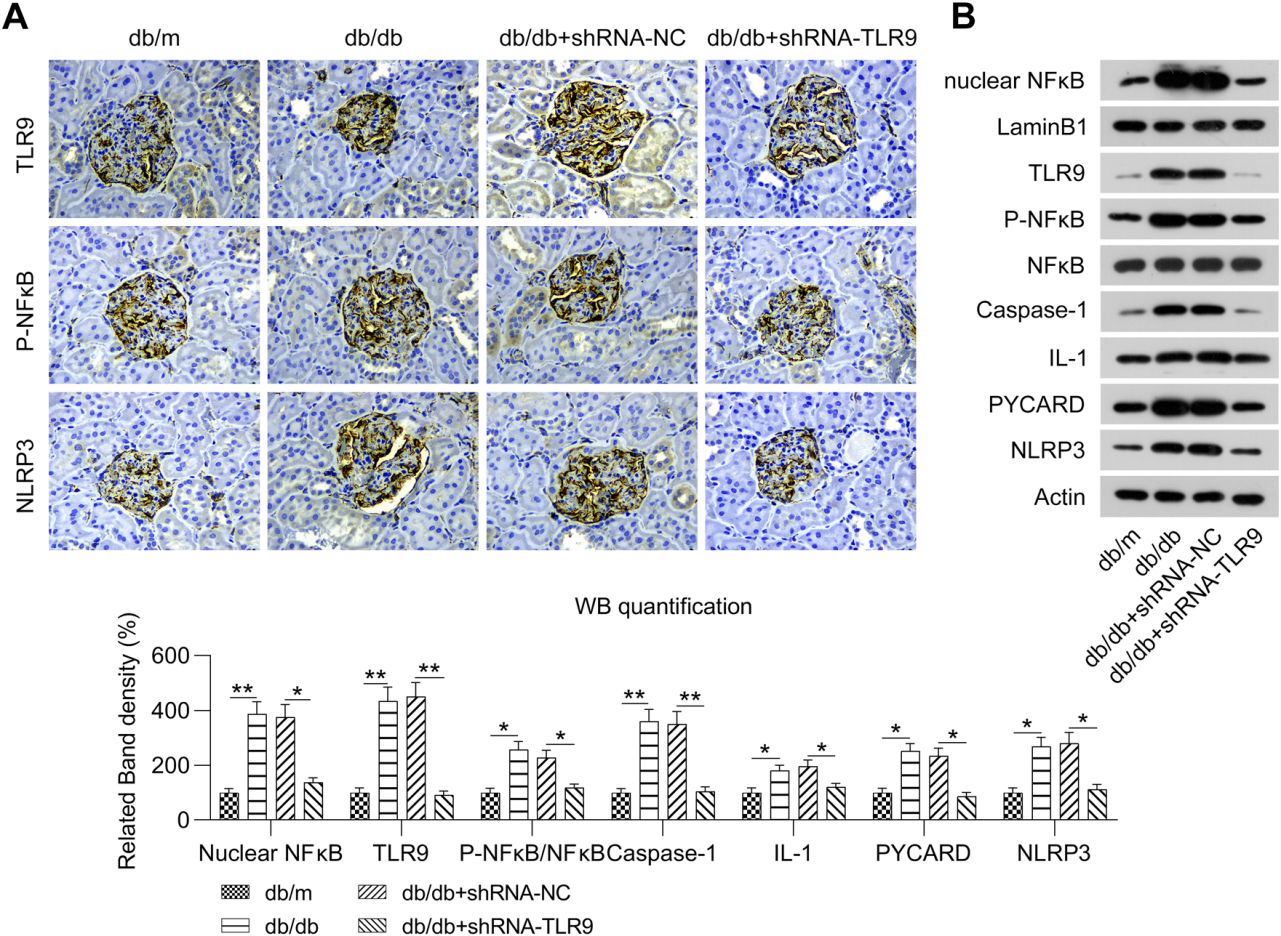
Effect of TLR9 knockdown on the NF-kB-NLRP3 inflammasome pathway in renal tissues of experimental mice (db/db). Experimental mice (db/db) were administered lentiviral-shRNA-NC or lentiviral-shRNA-TLR9 via tail vein injection. **A** Representative IHC image of TLR9, phosphor NF-kB, and NLRP3 antibodies in the glomeruli of mice (db/m) and mice with DN (db/db). **B** WB was conducted to determine the protein levels of TLR9, NF-kB, NLRP3, nuclear NF-kB, phosphor NF-kB, PYCARD, IL-1β, and cysteinyl aspartate-specific proteinase-1 in the renal tissues of mice (db/m) and mice with DN (db/db)

To elucidate the effect of TLR9 on DN, experimental mice (db/db) were administered lentiviral-shRNA-TLR9 via tail vein injection for 4 weeks to knockdown TLR9 expression. TLR9 expression in the kidneys of mice was confirmed to be downregulated using WB (Fig. 2A, B). Several DN parameters were determined in normal mice and db/db mice. Kidney and body weights were evidently higher in the NC-treated mice (db/db) with MD than in control mice (db/m) without MD. Further, kidney and body weights were remarkably decreased in the TLR9-knockdown experimental mice (db/db) than in the NC-treated experimental mice (db/db) (Table 1).

**Table 1:** TLR9 knockdown function on the progression of DN in experimental mice (db/db)

Indices	db/m	db/db	db/db + shRNA-NC	db/db + shRNA-TLR9
Body weight, g	21.31 ± 2.59	43.25 ± 5.06*	43.06 ± 6.01	32.62 ± 3.63^#^
Kidney weight, g	0.15 ± 0.03	0.25 ± 0.05*	0.24 ± 0.06	0.19 ± 0.03^#^
Blood glucose, mmol/L	6.99 ± 0.88	28.39 ± 3.65**	27.12 ± 3.85	19.63 ± 2.50^#^
SCR, μmol/L	35.12 ± 7.39	50.25 ± 5.20*	51.85 ± 5.75	41.05 ± 4.06^#^
BUN, mmol/L	8.70 ± 1.05	9.45 ± 0.96	9.40 ± 0.89	9.36 ± 0.93
Urinary albumin, μg/24 h	45.12 ± 13.75	693.75 ± 158.69***	720.15 ± 185.10	275.52 ± 56.82^##^

db/m, nondiabetic mice; db/db, diabetic mice (mock-treated); db/db + shRNA-NC, experimental mice (db/db) treated with lentiviral-shRNA-NC at 100 mg per kg body weight; db/db + shRNA-TLR9, experimental mice (db/db) treated with lentiviral-shRNA-TLR9 at 100 mg per kg body weight

Values are expressed as mean ± SD. SCR, serum creatinine; BUN, blood urea nitrogen. (*P < 0.05, **P < 0.01, ***P < 0.001 vs. db/m group; ^#^*P* < 0.05, ^##^*P* < 0.01 vs. db/db + shRNA-NC group)

According to a previous report, experimental mice (db/db) showed high blood sugar levels [22]. Therefore, blood sugar concentrations were significantly higher in db/db mice than in db/m mice. In the TLR9-knockdown experimental mice (db/db), blood glucose levels were mitigated (Table 1). Experimental mice (db/db) were also found to have large amounts of proteinuria. The UARE of NC-treated experimental mice (db/db) was significantly higher than that of other mice (db/m). shRNA-TLR9 administration significantly reduced proteinuria compared with that of NC-treated experimental mice (db/db). The glomerular filtration marker (SCR level) was also remarkably lower in the TLR9 gene knockout group than in the NC treatment group. The BUN level was not found to be influenced by TLR9 knockdown compared with that of the NC treatment (Table 1).

### Influence of TLR9 knockdown on the activation of NF-kB-NLRP3 inflammasome pathway in experimental mice (db/db)

A previous study indicated that the activated NF-kB-NLRP3 inflammasome pathway induces inflammation in DN [23], and TLR9 is located upstream of this pathway [24]. Therefore, we determined whether the NF-kB-NLRP3 inflammasome pathway is modulated via TLR9. IHC analyses revealed that the protein levels of phospho-NF-kB and NLRP3 were significantly increased in the renal tissues of experimental mice (db/db) (Fig. 2A), a finding confirmed via WB analysis (Fig. 2B). Furthermore, in the TLR-knockdown experimental mice (db/db), the expression levels of phospho-NF-kB and NLRP3 were significantly downregulated compared with that of NC-treated mice (Fig. 2A, B). We also determined the nuclear NF-kB levels and expression of other components of the NLRP3 inflammasome, namely, PYCARD, cysteinyl aspartate -specific proteinase-1, and interleukin-1β in the renal tissue of mice. Nuclear NF-κB, PYCARD, cysteinyl aspartate-specific proteinase-1, and interleukin-1β levels were found to be significantly increased in the renal tissue of experimental mice (db/db), while TLR9 knockdown decreased the levels of these proteins (Fig. 2B). Such findings indicate that the activation of the NF-kB-NLRP3 inflammasome pathway is regulated by TLR9 in DN mice.

### Effects of TLR9 knockdown on renal histology and collagen IV and fibronectin expression

**Fig. 3:**
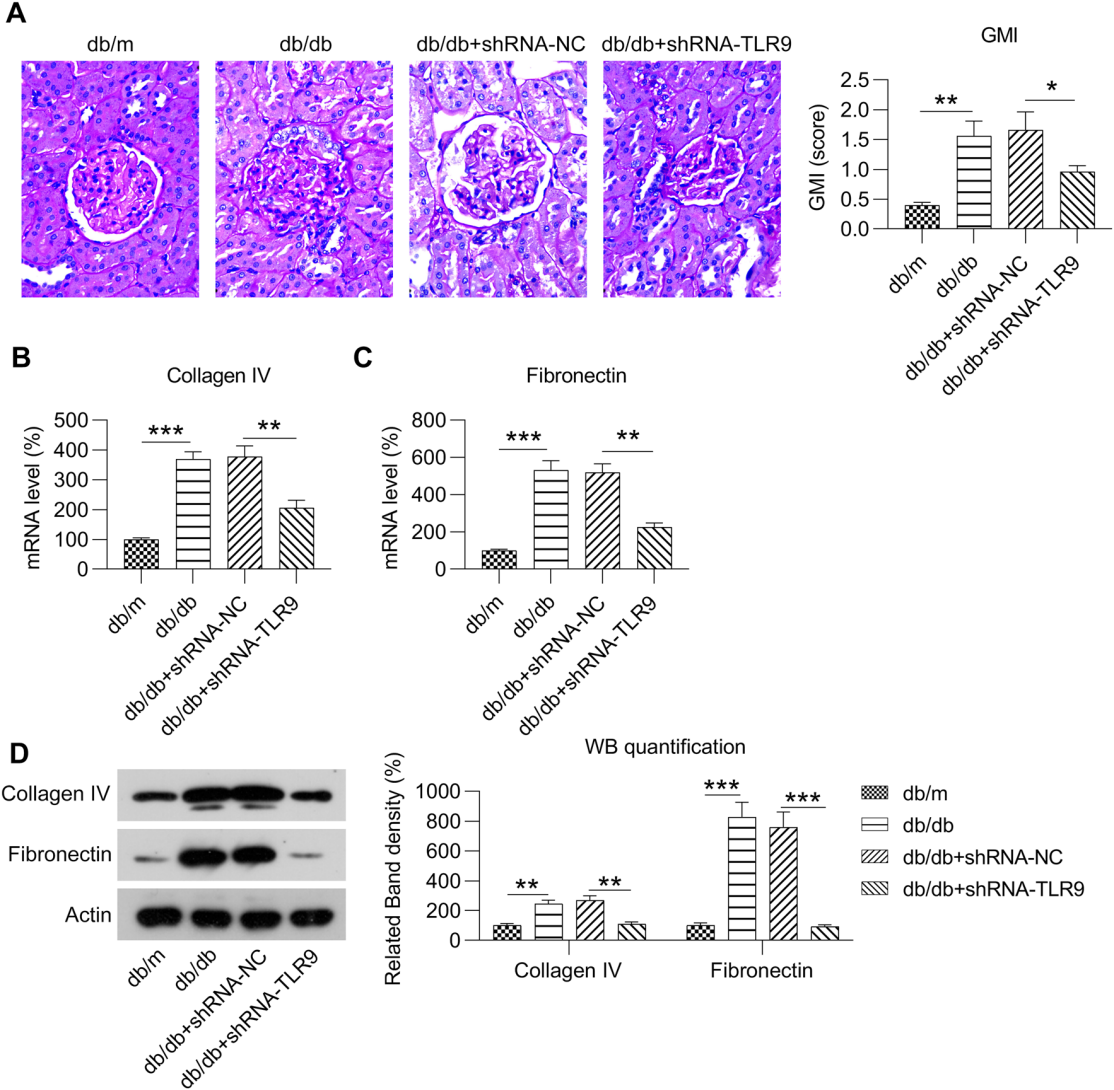
Effect of TLR9 knockdown on glomerular expansion and the expression levels of fibronectin and collagen IV in renal tissue from experimental mice (db/db). Experimental mice (db/db) were administered lentiviral-shRNA-NC or lentiviral-shRNA-TLR9 via tail vein injection. **A** Representative image of PAS-stained renal cortical slices from mice (db/m), experimental mice (db/db), NC-treated experimental-mice (db/db), and TLR9 knockdown experimental mice (db/db). GMI scores calculated based on the analysis of six glomeruli per mice. **B** RT-PCR was conducted to determine the mRNA expression levels of fibronectin and collagen IV in renal tissues. **C** WB was conducted to determine the protein levels of fibronectin and collagen IV in renal tissues. The data are representative of the results of three independent experiments, and the data are presented as means ± S.E.M (*P < 0.05, **P < 0.01, ***P < 0.001)

We evaluated renal pathogenesis in a mouse model by PAS staining and found evidence of mesangial matrix expansion in the glomeruli of NC-treated experimental mice (db/db) (Fig. 3A). Instances of PAS-positive mesangial matrix areas were also significantly higher in the NC- treated experimental mice (db/db) than in the other mice (db/m). These phenotypic changes were partially alleviated by TLR9 knockdown. The GMI score was significantly lower in TLR9 knockdown experimental mice (db/db) than in the NC-treated experimental mice (db/db) (Fig. 3A), indicating that TLR9 knockdown inhibits renal pathological changes.

An increase in the expression levels of fibronectin and collagen IV is a typical representation of DN [25]. Fibronectin and collagen IV levels were found to be promoted in the glomerular mesangial areas in experimental mice (db/db) compared with those of control mice (db/m) (Fig. 3B–D). Furthermore, proteins were expressed at lower levels in the glomerulus of TLR9 knockdown experimental-mice (db/db) than in the NC-treated experimental mice (db/db), as shown using RT-PCR and WB. Such findings suggest that TLR9 knockdown repressed the expression of fibronectin and collagen IV during DN.

### Influence of TLR9 on the NF-kB-NLRP3 inflammasome pathway in HG-treated MCs

**Fig. 4:**
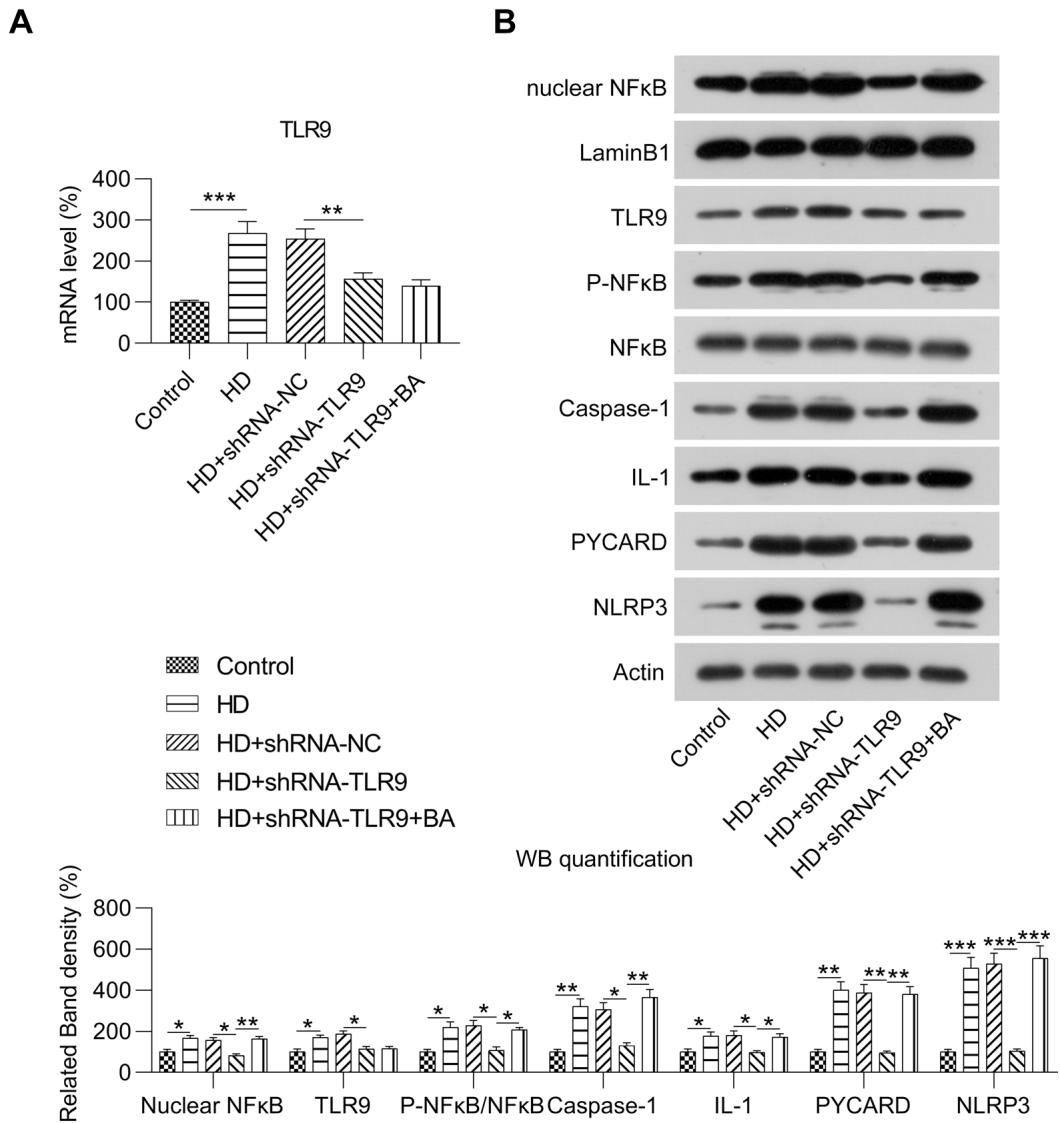
Role of TLR9 knockdown and BA treatment in the NF-kB-NLRP3 inflammasome pathway of HG-induced MCs. HG-treated MCs were transfected with shRNA-TLR9 or shRNA-TLR9 for 36 h and then treated the 1 µm for twelve hours. **A** RT-PCR was conducted to determine the mRNA expression level of TLR9 in MCs. **B** WB was conducted to determine the protein levels of TLR9, NF-kB, NLRP3, nuclear NF-kB, phosphor NF-kB, PYCARD, interleukin-1β, and cysteinyl aspartate-specific proteinase-1 in MCs. The data are representative of the results of three independent experiments, and the data are presented as means ± S.E.M (**P < 0.01, ***P < 0.001)

To explore the influence of TLR9 on NF-kB-NLRP3 inflammasome activation, we analyzed NF-kB activation and NLRP3 protein expression levels along with the formation of cysteinyl aspartate-specific proteinase-1 and interleukin-1 beta in MCs. Transfection of shRNA-TLR9 contributed to the downregulation of TLR9 expression in HG-treated MCs (Fig. 4A, B). However, nuclear NF-kB, phospho-NF-kB, and NLRP3 protein expression levels and PYCARD, cysteinyl aspartate specific proteinase-1, and interleukin-1β expression levels were reduced in MCs after TLR9 knockdown (Fig. 4B). To determine the influence of NF-kB during TLR9 knockdown in MCs, the activator betulinic acid (BA) was used to treat the cells. WB revealed that nuclear NF-kB and phosphorylated NF-kB levels were increased upon BA administration, indicating the successful activation of NF-kB in MCs with TLR9 knockdown. In addition, we observed an increase in the expression of the NLRP3 inflammasome in BA-treated TLR-knockdown MCs (Fig. 4B). These findings suggested that TLR9-regulated NLRP3 inflammasome in HG-treated MCs was NF-kB-dependent.

### Effect of the TLR9-NF-kB-NLRP3 inflammasome axis on inflammation and apoptosis in HG-treated MCs

**Fig. 5:**
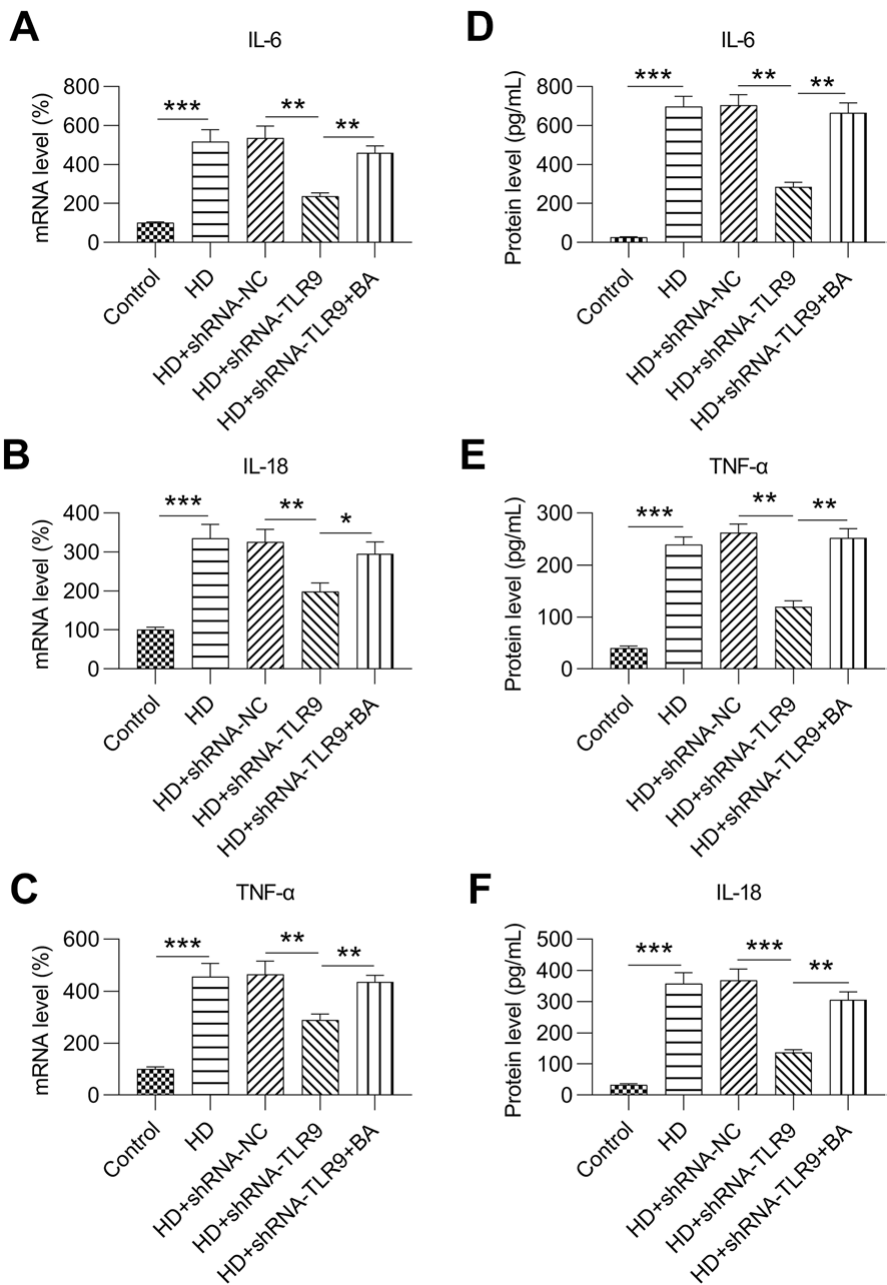
Role of TLR9 knockdown and BA treatment on inflammation in HG-induced MCs. HG-treated MCs were transfected with shRNA-TLR9 or shRNA-TLR9 for 36 h. Thereafter, processed the 1 µm for twelve hours. (A) Real-time PCR was performed to determine the mRNA expression levels of IL-6, IL-18, and TNF-alpha in MCs. (B) ELISA was performed to assess the released protein levels of IL-6, IL-18, and TNF-α in the supernatant of cultured MCs. The data are representative of the results of three independent experiments, and the data are presented as means ± S.E.M (*P < 0.05, **P < 0.01, ***P < 0.001)

A previous study revealed that high-glucose treatment induced inflammation and apoptosis in MCs [26, 27]. Herein, we sought to determine whether inflammation and apoptosis are induced in MCs treated with high levels of glucose. Expression of pro-inflammatory cytokines, IL-6, IL-18, and tumor necrosis factor-α, was upregulated in HG-treated MCs compared with that of control MCs according to both RT-PCR and ELISA (Fig. 5A, B). TLR9 knockdown ameliorated the high expression of cytokines in the HG-treated MCs, while BA administration counteracted the influence of TLR9 knockdown on inflammatory cytokines in MCs with HG treatment and TLR9 knockdown (Fig. 5A, B). These findings indicate that the TLR9-NF-kB-NLRP3 inflammasome axis effectively regulates inflammation in HG-treated MCs.

**Fig. 6:**
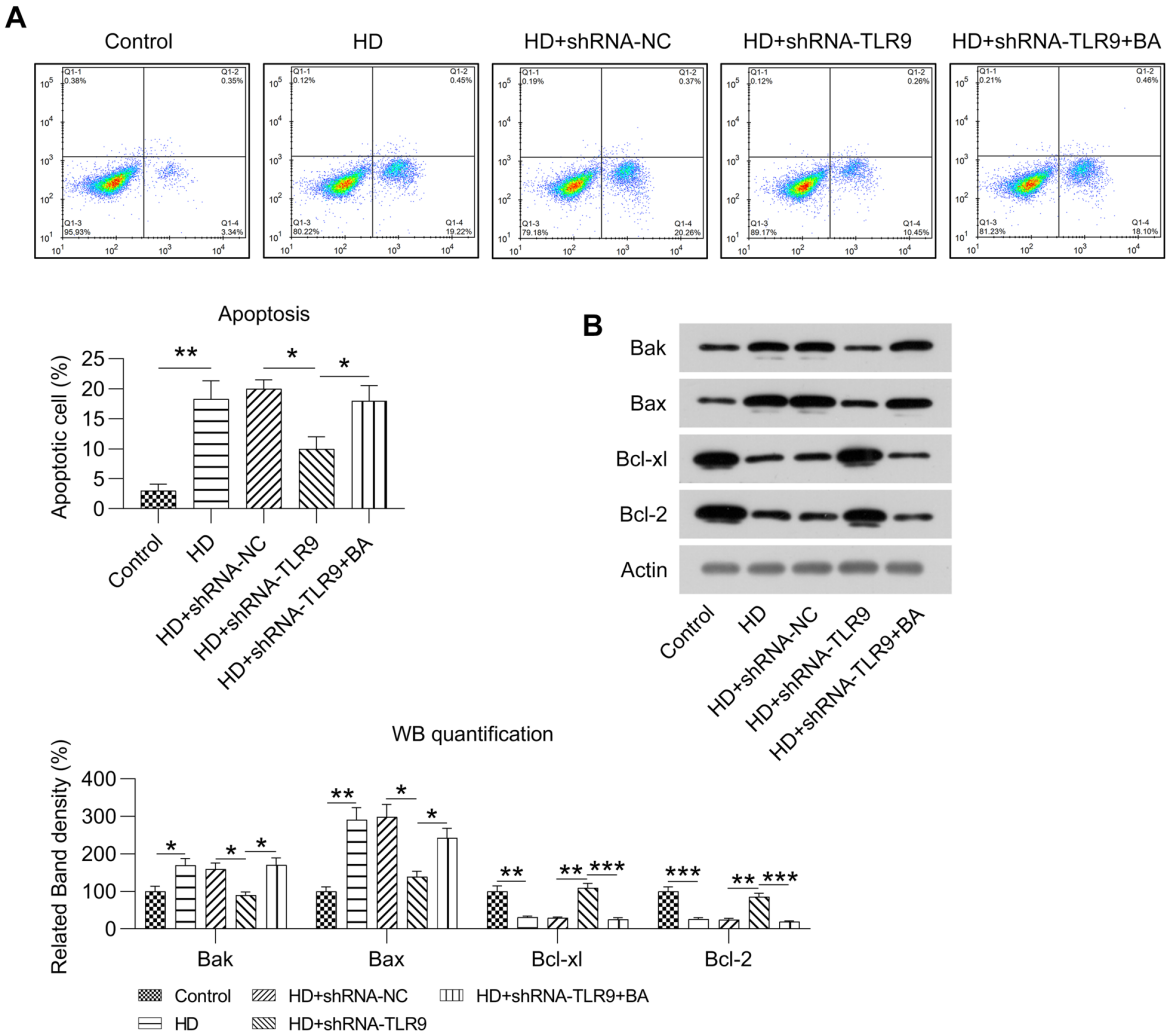
Role of TLR9 knockdown and BA treatment in the apoptosis of HG-induced MCs. HG-treated MCs were transfected with shRNA-TLR9 or shRNA-TLR9 for 36 h and then treated the 1 µm for twelve hours. **A** Cellular death was detected using FC with Annexin V-FITC as well as PI staining. **B** WB revealed the protein levels of Bcl-xl, Bcl-2, and Bak in MCs. The data are representative of the results of three independent experiments, and the data are presented as means ± S.E.M (*P < 0.05, **P < 0.01)

FC with Annexin V-FITC and PI staining was performed to evaluate apoptotic cell quantity. The proportion of apoptotic cells was significantly increased by HG treatment; however, this increase was less in the TLR9-knockdown HG-treated MCs (Fig. 6A). BA treatment also led to a higher rate of apoptosis in treated cells than in non-treated MCs (Fig. 6A). WB was carried out to detect Bcl-2, Bcl-xl, Bax, and Bak levels in cell strains subjected to various treatments. HG reduced Bcl-xl and Bcl-2 levels and increased Bak and Bax levels in MCs. However, TLR9 knockdown upregulated Bcl-2 and Bcl-xl levels and downregulated Bax and Bak levels in HG-treated MCs. The administration of BA counteracted the effect of TLR9 knockdown on these apoptotic markers in MCs (Fig. 6B). These data suggest that TLR9 knockdown suppressed apoptosis in HG-treated MCs through NF-kB activation.

## Discussion

This study sought to reveal the influence of TLR9 dysregulation on DN in experimental mice (db/db mice) and explore the potential mechanisms affecting HG-treated MCs. In this study, we first discovered that TLR9 expression levels were upregulated in PBMCs from patients with DN, kidney tissues from experimental mice (db/db), and HG-treated MCs, compared with that of their respective controls. Knockdown of TLR9 expression directly restrained NF-kB and suppressed inflammatory molecular expression in vitro and in vivo, mediated by the NLRP3 inflammasome. TLR9 is related to the development of fibrosis and microalbuminuria in DN mice. Such findings offer a novel model for the constitutive activation of the NLRP3 inflammation pathway in DN, mediated by NF-κB.

Proteinuria development, a characteristic of DN, is commonly accompanied by a progressive decline in renal function. Urinary albumin excretion is considered the earliest mark of glomerular injury in dominant DN. The injury, which is anatomically characterized by thickening of the basilar membrane and ME, can be revealed anatomically as an increase in the area of the PAS-positive mesangial matrix, which results from the summation of ECM proteins [28]. During this investigation, we discovered that the increased urinary albumin excretion, mesangial matrix expansion, as well as upregulated type IV collagen and fibronectin expression in the experimental db/db mice were inhibited after TLR9 gene knockdown, compared with that of the NC group. Additionally, after the TLR9 gene was knocked out, body/kidney weight and SCR expression levels changed significantly. These findings suggest that TLR9 is important in advanced stages of DN in experimental mice (db/db).

A previous investigation revealed that in an intestinal inflammation model, TLR9 deficiency resulted in the over-activation of NF-κB and NLRP3 inflammasome signaling pathways, resulting in excessive interleukin-1β secretion, which is inconsistent with our findings. Our in vivo and in vitro data both suggest that TLR9 knockdown caused the de-activation of NF-kB and NLRP3 inflammasome, as revealed by the reduced levels of phosphorylated NF-kB and nuclear NF-kB, as well as the downregulated expression of NLRP3, IL-1β, and cysteinyl aspartate-specific proteinase-1. Our data indicate a novel regulatory axis in TLR9-regulated NF-kB and NLRP3 inflammasomes in DN development.

Diabetic animals showed significant NF-ĸB activation in their kidneys, as revealed by the increased phosphorylation of IĸB and subsequent increased nuclear translocation of NF-kB. The decrease in the activity of this transcription factor was related to the improvement of proteinuria as well as renal structural damage in CKD models [29]. Wang et al. demonstrated that in a DN rat model, both TLRs and NF-kB were upregulated and activated. Umbelliferae, a family of flowering plants, could reverse these phenotypic changes and significantly mitigates renal histopathological effects [30]. Hassan et al. found that *Ganoderma lucidum*, a mushroom commonly used in traditional Asian medicine, downregulated TLR4 and NF-κB pathway activation and remarkably mitigated kidney-related damage by correcting deteriorative renal effects and improving oxidative stress in DN rats [31]. This study found that TLR9 is an upstream modulator of NF-kB. Knockdown of TLR9 expression as well as usage of BA in HG-treated MCs indicated that the prohibition of IĸB phosphorylation and the reduction of nuclear NF-kB localization affected inflammation as well as regulated apoptosis.

The renal protection functions of TLR9 depletion may be caused by the inhibition of NLRP3 inflammasome activation, and the production of interleukin-1 beta and cysteinyl aspartate specific proteinase-1 may be affected by the regulatory role of TLR9 in NF-kB activation [32, 33]. TLR9 deficiency has been demonstrated to reduce NLRP3 inflammasome activation and interleukin-1 beta production in NKG2D-mediated intestinal inflammation induced by *Salmonella* [33]. Interleukin-1 beta and cysteinyl aspartate-specific proteinase-1, which are both key components of the NLRP3 inflammasome, participate in DN development [34, 35]. The NLRP3 inflammasome is a significant factor in inflammation and tissue damage during acute kidney injury (AKI), CKD [36], renal inflammation, and fibrosis [37]. The role of TLRs as well as NLRP3 inflammasomes in the model of renal TIN has been well documented. Further, allopurinol has been found to downregulate the expression of a single component of the inflammasome pathway and to reduce this damage [38]. Taken together, these results suggest that the TLR9-related NLRP3 inflammasome is a possible treatment target for DN.

## Conclusions

In conclusion, we found that the downregulation of TLR9 expression attenuated the expression of inflammation and apoptosis factors through the NF-kB and NLRP3 inflammasome pathways in HG-treated MCs and mice (db/db) with DN. Furthermore, knockout of the TLR9 gene could inhibit the renal function of mice (db/db) with DN and reduce glomerular damage. Such findings offer novel insights into the renal protection mechanisms related to TLR9 and NF-kB/NLRP3, which may lead to the development of new treatment strategies for DN.

## Data Availability

The datasets used and/or analyzed during the current study are available from the corresponding author upon reasonable request.
